# Oocytes with aggregates of smooth endoplasmic reticulum may not affect reproductive outcomes in split IVF-ICSI insemination: a retrospective study

**DOI:** 10.3389/fendo.2025.1567066

**Published:** 2025-04-29

**Authors:** Yejuan Li, Jiajia Hu, Hui Lu, Zhiyong Lu, Jingjing Zhong, Lisen Shi

**Affiliations:** ^1^ Reproductive Medical Center, Hainan Women and Children’s Medical Center, Haikou, China; ^2^ Medical Genetics and Prenatal Diagnosis, Haikou Maternal and Child Health Hospital, Haikou, Hainan, China

**Keywords:** split IVF-ICSI, oocyte, SERa, embryological outcomes, clinical and neonatal outcomes

## Abstract

**Objective:**

To investigate the impact of smooth endoplasmic reticulum aggregates (SERa) in oocytes on embryological outcomes and clinical and neonatal outcomes during split IVF-ICSI cycles.

**Methods:**

A retrospective analysis was conducted using clinical data from January 2020 to December 2023 at the Reproductive Medicine Center of Hainan Women and Children’s Medical Center. Patients were divided into SERa+ and SERa- cycles based on the visibility of SERa after the removal of cumulus cells. Basic patient characteristics, embryological outcomes, clinical and neonatal outcomes were compared between the two groups.

**Results:**

Compared to the SERa- cycles, the SERa+ cycles showed significantly higher levels of E_2_ on the day of hCG administration (P<0.01) and a significantly increased number of retrieved oocytes (P<0.01). In terms of embryological outcomes, the total D3 high-quality embryo rate was significantly higher in the SERa+ cycles (P<0.01). There was a significant increase in the D3 high-quality embryo rate for ICSI, but no difference in the D3 high-quality embryo rate for IVF. No significant differences were observed between the SERa+ and SERa- cycles in terms of βhCG positivity rate, clinical pregnancy rate, implantation rate, early miscarriage rate, live birth rate, preterm birth rate, newborn height, and weight (P>0.05). No congenital birth defects were found in either group.

**Conclusion:**

The occurrence of SERa in split IVF-ICSI cycles may be associated with increased E_2_ levels on hCG day, and the presence of SERa does not appear to affect *in vitro* fertilization, embryological, clinical, or neonatal outcomes.

## 1 Introduction

In assisted reproductive technology, the quality of the oocyte directly influences the quality of the embryo and its subsequent developmental potential (1, 2). The smooth endoplasmic reticulum aggregate (SERa), first identified in 1997 (3), is a cytoplasmic anomaly characterized by a central, round, transparent, and flat disc within the oocyte’s cytoplasm. SERa has attracted considerable attention in reproductive medicine. The incidence of SERa varies widely, with reported rates ranging from 4.0% to 23.1% in cycles and 17.6% to 34.4% in individual oocytes (4). The release of calcium from the SER plays a critical role in oocyte maturation, fertilization, and early embryonic development (5, 6). Although the precise mechanisms underlying SERa formation remain unclear, ongoing research and data collection are essential for understanding its impacts and mechanisms. In 2004, a case was reported where a baby diagnosed with Beckwith-Wiedemann syndrome was born following a cycle involving SERa+ oocytes (7). Subsequent studies have indicated a significant decrease in live birth rates in cycles with SERa+ oocytes, along with a relatively higher incidence of congenital anomalies (8–10). Given these potential negative effects, the 2011 Istanbul Consensus recommended against using SERa+ oocytes (11). However, other studies have not observed an increased risk of congenital anomalies in embryos derived from SERa+ oocytes, nor have they found reduced pregnancy rates (12–14). It is reported that only 14% of centers discard SERa+ oocytes (15). Due to these inconsistent findings, the revised Vienna consensus by Alpha/ESHRE reconsidered this recommendation in 2017, advising a case-by-case approach (16). Therefore, in clinical IVF practice, the lack of consistent guidelines has led to varying attitudes among clinicians and embryologists regarding the handling of SERa+ oocytes, highlighting the urgent need for extensive clinical data to inform decision-making in embryo transfer.

Currently, clinical studies on SERa are expanding, primarily focusing on either intracytoplasmic sperm injection (ICSI) or conventional *in vitro* fertilization (IVF) cycles involving SERa-positive oocytes. However, there is a paucity of research investigating the impact of SERa in split IVF-ICSI cycles on embryonic development and clinical outcomes. This study aims to conduct a comprehensive analysis of clinical data from patients with SERa-positive oocytes undergoing split IVF-ICSI cycles at the Hainan Women and Children’s Medical Center between January 2020 and December 2023. By exploring the effects of SERa on early embryological, clinical, and neonatal outcomes, this research seeks to provide scientific guidance for managing SERa-positive oocytes in assisted reproductive treatments while optimizing embryo transfer strategies. Ultimately, this study aspires to enhance both the success rate and safety of clinical applications.

## 2 Materials and methods

### 2.1 Patients and study design

**Figure 1:**
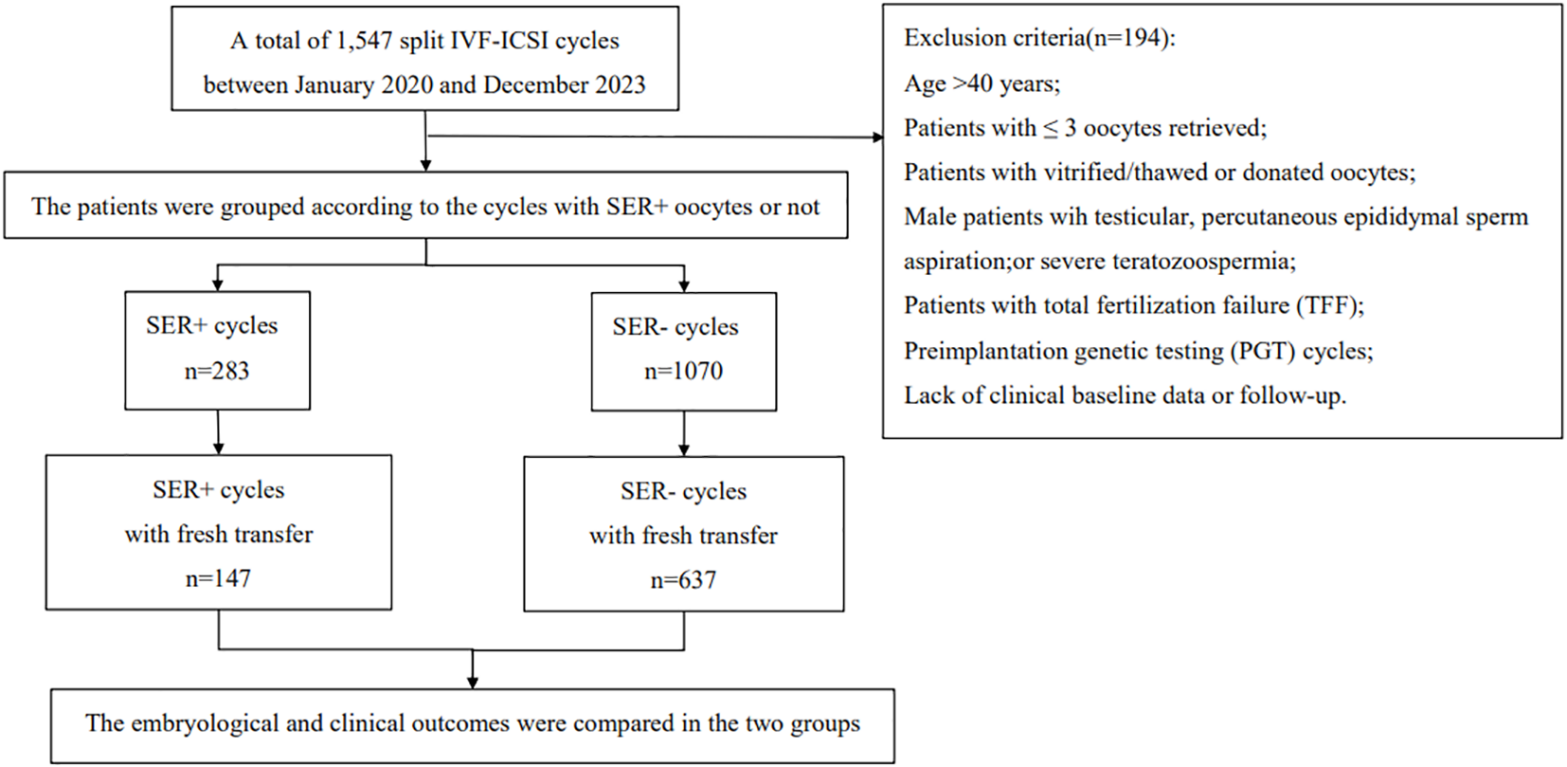
Flow chart of the study.

This study selected infertile couples undergoing *in vitro* fertilization-embryo transfer (IVF-ET) treatment at the Hainan Provincial Women’s and Children’s Medical Center from January 2020 to December 2023 as research subjects. Inclusion criteria: suitability for split IVF‐ICSI treatment; fresh oocyte retrieval cycles; age ≤ 40 years. Exclusion criteria: age > 40 years; patients with≤ 3 oocytes retrieved; patients utilizing vitrified/thawed or donated oocytes; male patients with testicular issues, percutaneous epididymal sperm aspiration, or severe teratozoospermia;patients experiencing total fertilization failure (TFF); preimplantation genetic testing (PGT) cycles; and those lacking clinical baseline data or follow-up. According to the presence or absence of SERa in oocytes, participants were divided into two groups: SERa+ cycles (at least one oocyte testing positive for SERa) and SERa- cycles (no oocytes with SERa). The flow chart illustrating patient inclusion in this study is presented in **Figure 1**.

### 2.2 Research methodology

#### 2.2.1 Ovulation induction and oocyte retrieval

All patients underwent ovulation induction and follicular monitoring in accordance with the standard clinical protocols established at our center. The dosage of gonadotropins was tailored to each patient, taking into consideration factors such as age, body mass index (BMI), antral follicle count (AFC), and their response to previous ovarian stimulation cycles. Oocyte retrieval was conducted 34–37 hours post-triggering, once dominant follicles reached a diameter of 17–18 mm, utilizing transvaginal ultrasound guidance for precision in the procedure, during which the oocytes were meticulously recorded.

#### 2.2.2 ICSI, IVF fertilization, embryo culture, and morphological observation

The retrieved cumulus-oocyte complexes (COCs) in ICSI insemination were maintained in G-IVF PLUS medium (Vitrolife, Sweden) for 3~4 hours prior to cumulus cell removal. ICSI was conducted 1 to 2 hours after denudation, with careful attention taken to avoid injecting sperm into the SERa. Only MII oocytes were utilized for ICSI. Comprehensive records of SERa+ oocytes were meticulously maintained during the ICSI procedure and subsequently entered into the system. IVF insemination occurred 3 to 4 hours following oocyte retrieval, ensuring that the concentration of progressively motile sperm (PR) was controlled at a range of 100,000-150,000/ml. The remaining COCs were fertilized by IVF using overnight fertilization and degranulated 16~17 hours after insemination. Fertilization assessment took place approximately 16 to 18 hours later under a magnification of 400×using an inverted microscope, focusing on the identification of pronuclei. Embryos were cultured *in vitro* for a duration of 3 to 7 days under controlled conditions of 37°C, with an atmosphere comprising 5% O2 and 6% CO2 in Vitrolife culture media. Observations and detailed records regarding fertilization outcomes, subsequent embryonic development, and pregnancy results post-transfer were systematically documented.

#### 2.2.3 SERa evaluation

**Figure 2:**
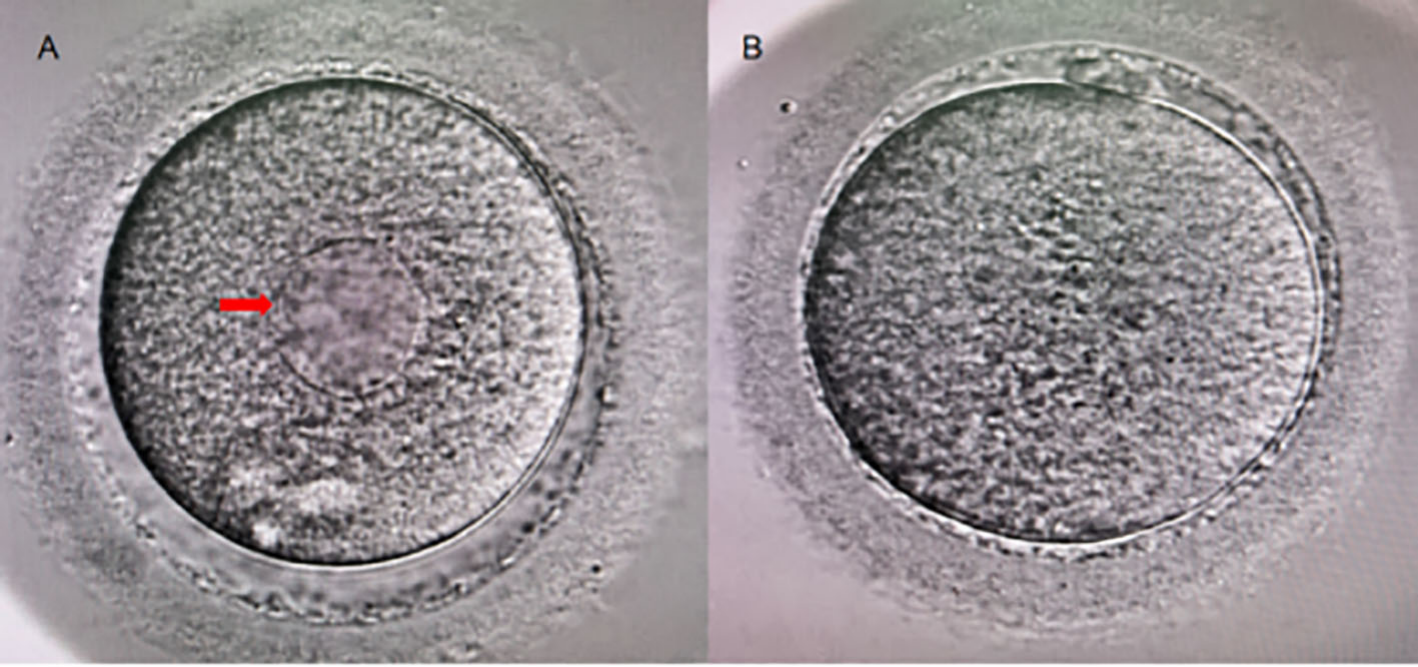
Human Metaphase II Oocytes (400×). **(A)** A metaphase II oocyte exhibiting SERa (indicated by the red arrow); **(B)** A normal metaphase II oocyte.

ICSI insemination was conducted 1–2 hours post oocyte denudation, during which SERa were also evaluated. For IVF fertilization, SER observation was performed concurrently with pronuclear assessment on the following day after denudation. Oocytes were examined under high magnification (400×). Normal oocytes exhibit a uniform distribution of cytoplasm. The presence of large, round, flat, semi-transparent discoid structures within the cytoplasm indicates the occurrence of SERa, as illustrated in **Figure 2** with a red arrow (**Figure 2**).

#### 2.2.4 Embryo quality assessment

Embryo assessment time points were determined according to the standardized criteria outlined in the Istanbul Consensus Protocol, while prokaryotic scoring was performed utilizing the Scott-Z assessment. Embryos were comprehensively evaluated at the cleavage stage following our center’s established protocol, which takes into account embryo morphology, developmental rate, and blastomere count. Scoring of blastocysts was conducted according to the Gardner system.

#### 2.2.5 Primary observational indicators

The formulas for laboratory and clinical observational indicators are presented in **Tables 1** and **2**, respectively (**Tables 1**, **2**).

**Table 1:** Laboratory observational indicator.

Term	Formula
MII oocyte rate	Total number of MII oocytes/Total number of retrieved oocytes ×100%
Total fertilization rate	Total number of fertilized oocytes/Total number of MII oocytes ×100%
Normal fertilization rate	Number of normally fertilized oocytes/Total number of MII oocytes ×100%
Cleavage rate	Number of cleaved embryos/Number of fertilized oocytes×100%
High-quality Day 3 embryo rate	High-quality Day 3 embryos/Number of normally fertilized cleaved embryos ×100%
Blastocyst formation rate	Stage 2 and above blastocysts/Total number of cleavage-stage embryos cultured for blastocyst × 100%
High-quality blastocyst rate	Number of high-quality blastocysts/Number of stage 2and above blastocysts × 100%
Available blastocyst rate	Number of usable blastocysts/Number of stage 2 and above blastocysts × 100%

(i) High-quality Day 3 embryo: Normally fertilized and has 7–9 cells with ≤10% fragmentation on Day 3.

(ii) High-quality blastocyst: Stage 3 and above blastocysts with inner cell mass and trophectoderm cells not containing grade C.

(iii) Available blastocyst: Stage 3 and above blastocysts where neither the inner cell mass nor trophectoderm cells simultaneously contain grade C.

**Table 2:** Clinical observational indicators.

Term	Formula
β-hCG positivity rate	Total number of β-hCG positive individuals/Total number of transfer patients ×100%
Clinical pregnancy rate	Total number of clinical pregnancies/Total number of transfer patients ×100%
Implantation rate	Number of implanted embryos/Number of transferred embryos ×100%
Early miscarriage rate	Number of early miscarriages/Total number of clinical pregnancies ×100%
Live birth rate	Number of live births/Number of transfer cycles ×100%
Preterm birth rate	Number of preterm deliveries/Number of transfer cycles ×100%

#### 2.2.6 Statistical methods

The one-sample Kolmogorov-Smirnov test was employed to evaluate the normality of continuous data. Continuous variables were expressed as mean ± standard deviation (SD) when they followed a normal distribution. Differences in continuous variables were analyzed using the t-test. Mann-Whitney U test was applied for non-normally distributed data. Categorical variables were presented as percentages, with differences assessed using either the chi-squared test or Fisher’s exact test, depending on appropriateness. Statistical analyses were conducted using the Statistical Program for Social Sciences (SPSS Inc., Version 27.0, Chicago, IL, USA). A p-value of less than 0.05 in a two-tailed test was deemed statistically significant.

## 3 Results and analysis

This study collected data from 1,547 fresh oocyte retrieval cycles that underwent split IVF-ICSI at the Hainan Provincial Women’s and Children’s Medical Center between January 2020 and December 2023. Among these cycles, 309 were identified as SERa+, resulting in a SERa positivity rate of 19.97%. After applying the inclusion and exclusion criteria, a total of 1,353 split IVF-ICSI cycles (including 283 SERa+ and 1,070 SERa- cycles) were included in the final analysis. Among SERa+ cycles, 588 SERa+ oocytes were identified, with 34 (5.8%) derived from conventional IVF and 554 (94.2%) from ICSI. Each SERa+ cycle contained an average of 2.08 ± 1.74 SERa+ oocytes. Notably, SERa+ oocytes represented a substantial proportion (13.73%, 588/4283) of all retrieved oocytes.

### 3.1 Comparison of general clinical data between the two groups

As summarized in **Table 3**, no statistically significant differences were observed between the two groups regarding age, duration of infertility, type of infertility, body mass index, and baseline levels of FSH, LH, E_2_, P, and AMH (P > 0.05). In terms of ovulation induction protocols, there were no differences noted in LH and P levels or the number of follicles on the day of hCG administration (P > 0.05). However, the levels of E_2_ on the day of hCG administration were significantly elevated in the SERa+ cycles (P < 0.01). Additionally, the average E_2_ level per oocyte on hCG day was notably higher in the SERa+ cycles (P < 0.05).With respect to medication usage, there were no significant differences between the groups concerning the total amount of Gonadotropin used or the duration for which Gn was administered (P > 0.05); nevertheless, the SERa+ cycles demonstrated a significant increase in the number of oocytes retrieved (P < 0.01).

**Table 3:** Comparison of general clinical data.

Item	SERa+ cycles (n=283)	SERa-cycles (n=1070)	*P*
Female age (years)	32.33 ± 3.82	31.96 ± 3.78	0.187
Duration of infertility (years)	4.72 ± 3.18	4.69 ± 2.95	0.629
Infertility types			0.155
Primary,% (n)	54.42 (154)	59.25 (634)	
Secondary,% (n)	45.58 (129)	40.75 (436)	
Female BMI (kg/m^2^)	21.95 ± 3.48	22.01 ± 6.78	0.918
Basal FSH level (IU/L)	6.38 ± 1.93	6.57 ± 2.12	0.109
Basal LH level (IU/L)	6.14 ± 3.55	6.13 ± 5.53	0.436
Basal E_2_ level (pg/mL)	67.61 ± 285.47	83.64 ± 487.48	0.213
Basal P level (ng/mL)	0.42 ± 1.84	0.40 ± 1.76	0.421
Basal AMH level (ng/mL)	4.14 ± 3.23	4.17 ± 3.09	0.743
E_2_ on hCG day (pg/mL)	3189.99 ± 1846.86	2764.97 ± 1515.84	0.001
Average E_2_ level per oocyte on HCG day	261.79 ± 119.82	243.70 ± 106.77	0.025
LH on hCG day (IU/L)	3.58 ± 9.12	3.29 ± 2.52	0.360
P on hCG day (ng/mL)	0.62 ± 0.35	0.58 ± 0.75	0.404
Mean no. of Follicle on hCG day	12.81 ± 5.52	12.06 ± 5.67	0.057
Mean no. of oocytes retrieved	15.50 ± 6.44	13.90 ± 6.35	0.001
Total gonadotrophins dose (U)	1865.70 ± 568.27	1849.26 ± 635.79	0.352
Duration of Gn (days)	9.88 ± 1.47	9.81 ± 1.87	0.106

### 3.2 Comparison of embryological outcomes between the two groups

In terms of embryological outcomes, as presented in **Table 4**, the SERa+ cycles exhibited no significant differences compared to the SERa- cycles regarding total fertilization rate, normal fertilization rate, cleavage rate, blastocyst formation rate, high-quality blastocyst rate, and available blastocyst rate (P > 0.05). However, the incidence of high-quality Day 3 embryos was significantly greater in the SERa+ cycles (P < 0.01).

**Table 4:** Comparison of embryological outcomes.

Item	SERa+ cycles (n=283)	SERa-cycles (n=1070)	*P*
Total fertilization rate,% (n)	82.83 (3474/4194)	82.26 (11643/14154)	0.406
Total normal fertilization rate,% (n)	70.43 (2954/4194)	70.92 (10038/14154)	0.549
Total cleavage rate,% (n)	97.58 (3390/3474)	97.67 (11372/11643)	0.754
Total high-quality Day 3 embryo rate,% (n)	42.68 (1227/2875)	38.37 (3742/9752)	0.001
Total blastocyst formation rate,% (n)	68.48 (1671/2440)	68.41 (5476/8005)	0.960
Total high-quality blastocyst rate,% (n)	68.04 (1137/1671)	67.71 (3708/5476)	0.811
Total available blastocyst rate,% (n)	83.90 (1402/1671)	84.02 (4601/5476)	0.909

### 3.3 Comparison of embryological outcomes in ICSI insemination between the two groups

As illustrated in **Table 5**, compared to the SERa- cycles, no significant differences were observed in the SERa+ cycles concerning the rate of MII mature oocytes, fertilization rate, normal fertilization rate, cleavage rate, blastocyst formation rate, high-quality blastocyst rate, and available blastocyst rate (P > 0.05). However, there was a significant increase in the rate of high-quality Day 3 embryo within the SERa+ cycles (P < 0.01).

**Table 5:** Comparison of embryological outcomes in ICSI insemination.

Item	SERa+ cycles (n=283)	SERa-cycles (n=1070)	*P*
MII oocyte rate,% (n)	96.04 (2161/2250)	94.71 (7067/7462)	0.011
ICSI fertilization rate,% (n)	86.81 (1876/2161)	87.18 (6161/7067)	0.660
ICSI normal fertilization rate,% (n)	78.90 (1705/2161)	79.92 (5648/7067)	0.299
ICSI cleavage rate,% (n)	98.67 (1851/1876)	98.46 (6066/6161)	0.587
ICSI-D3 high-quality embryo rate,% (n)	46.38 (774/1669)	40.11 (2215/5523)	0.001
ICSI blastocyst formation rate,% (n)	69.96 (983/1405)	71.03 (3136/4415)	0.459
ICSI high-quality blastocyst rate,% (n)	69.68 (685/983)	69.71 (2186/3136)	0.999
ICSI available blastocyst rate,% (n)	85.25 (838/983)	85.33 (2676/3136)	0.959

### 3.4 Comparison of embryological outcomes in IVF insemination between the two groups

In the context of IVF insemination, no significant differences were observed between the SERa+ and SERa- cycles regarding fertilization rate, normal fertilization rate, cleavage rate, high-quality Day 3 embryo rate, high-quality blastocyst rate, and available blastocyst rate (P > 0.05)(As illustrated in **Table 6**).

**Table 6:** Comparison of embryological outcomes in IVF insemination.

Item	SERa+ cycles (n=283)	SERa-cycles (n=1070)	*P*
IVF fertilization rate,% (n)	78.60 (1598/2033)	77.35 (5482/7087)	0.239
IVFnormal fertilization rate,% (n)	61.44 (1249//2033)	61.94 (4390/7087)	0.679
IVF cleavage rate,% (n)	96.31 (1539/1598)	96.79 (5306/5482)	0.342
IVF-D3 high-quality embryo rate,% (n)	37.56 (453/1206)	36.11 (1527/4229)	0.360
IVF blastocyst formation rate,% (n)	66.47 (688/1035)	65.18 (2340/3590)	0.458
IVF high-quality blastocyst rate,% (n)	65.70 (452/688)	65.04 (1522/2340)	0.785
IVF available blastocyst rate,% (n)	81.98 (564/688)	82.26 (1925/2340)	0.865

### 3.5 Comparison of clinical and neonatal outcomes between the two groups

There were 147 fresh transfer cycles in the SERa+ cycles and 637 fresh transfer cycles in the SERa- cycles. No significant differences were observed between the SERa+ and SERa- cycles regarding β-hCG positivity rate, clinical pregnancy rate, implantation rate, early miscarriage rate, live birth rate, preterm birth rate, as well as the heights and weights of newborns (P > 0.05). The SERa+ cycles recorded 68 live births while the SERa- cycles had 274 live births; notably, no congenital birth defects were identified in either cohort(As illustrated in **Table 7**).

**Table 7:** Comparison of clinical and neonatal outcomes.

Item	SERa+ cycles (n=147)	SERa-cycles (n=637)	*P*
Female age (years)	32.90 ± 3.83	32.35 ± 3.71	0.112
Average number of embryos transferred	1.17 ± 0.38	1.23 ± 0.42	0.123
proportions of transferred embryos			0.994
IVF-derived embryos,% (n)	52.91 (91/172)	52.94 (414/782)	
ICSI-derived embryos,% (n)	47.09 (81/172)	47.06 (368/782)	
β-hCG positivity rate,% (n)	58.50% (86/147)	60.44% (385/637)	0.709
clinical pregnancy rate,% (n)	54.42% (80/147)	53.06% (338/637)	0.784
implantation rate,% (n)	50.58% (87/172)	46.55% (364/782)	0.354
early miscarriage rate,% (n)	13.75% (11/80)	21.30% (72/338)	0.160
live birth rate,% (n)	41.50% (61/147)	39.56% (252/637)	0.709
preterm birth rate,% (n)	11.48% (7/61)	12.70% (32/252)	0.999
Total number of live infants (n)	68	274	–
Birth weight (kg)	2.99 ± 0.41	2.91 ± 0.57	0.182
Birth heights (cm)	48.93 ± 1.81	48.86 ± 2.08	0.797
Birth defects	0	0	–

### 3.6 Comparison of clinical and neonatal outcomes between the two groups in SERa+ cycles

Among the 147 SERa+ cycles, a total of 23 embryos derived from ICSI were transferred across 15 cycles. This included 16 embryos originating from SERa+ oocytes. No significant differences were observed between the SERa+ and SERa- oocytes in terms of β-hCG positivity rate, clinical pregnancy rate, implantation rate, early miscarriage rate, live birth rate, preterm birth rate, as well as the heights and weights of newborns (P > 0.05). The embryos derived from SERa+ oocytes resulted in 10 live births, while those from SERa- oocytes yielded 58 live births; notably, no congenital birth defects were identified in either cohort(As illustrated in **Table 8**).

**Table 8:** Comparative analysis of clinical and neonatal outcomes in SERa+ cycles.

Item	SERa+ Oocytes in SERa+ cycles (n=15)	SERa- Oocytes in SERa+ cycles (n=132)	*P*
Female age (years)	33.13 ± 3.20	32.87 ± 3.90	0.803
No. of embryos transferred (n)	23	149	–
Average number of embryos transferred	1.53 ± 0.52	1.13 ± 0.34	<0.001
IVF-derived embryos transferred proportion,% (n)	0 (0/23)	66.44 (99/149)	–
ICSI-derived embryos transferred proportion,% (n)	100 (23/23)	33.56 (50/149)	–
No. of SERa+ oocytes derived embryos transferred (n)	16	–	–
β-hCG positivity rate,% (n)	66.67% (10/15)	56.82% (75/132)	0.464
clinical pregnancy rate,% (n)	66.67% (10/15)	53.03% (70/132)	0.315
implantation rate,% (n)	52.17% (12/23)	50.34% (75/149)	0.870
early miscarriage rate,% (n)	10.00% (1/10)	15.71% (11/70)	1.000
live birth rate,% (n)	60.00% (9/15)	39.39% (52/132)	0.125
Total number of live infants (n)	10	58	–
Birth weight (kg)	3.04 ± 0.21	2.98 ± 0.44	0.494
Birth heights (cm)	49.70 ± 0.67	48.79 ± 1.91	0.141
Birth defects	0	0	–

## 4 Discussion

Fertilization of oocytes is a multifaceted process influenced by various factors, including the maturity of both the oocyte and sperm, as well as the vitality and fusion of genetic material. These elements are critical in assisted reproductive technology (ART). Certain infertility treatment cycles may experience low fertilization rates or even complete fertilization failure, with incidence rates ranging from 10% to 20%. Such challenges not only lead to repeated failures in subsequent assisted pregnancy attempts but also impose significant psychological and economic stress on individuals undergoing these treatments (17). The split IVF-ICSI technique plays a pivotal role in ART and serves as an effective strategy to mitigate low fertilization rates (18, 19). In our study, we observed the occurrence rate of SERa at 19.97% in split IVF-ICSI cycles; however, there is limited research on the impact of SERa on embryological, clinical, or neonatal outcomes within these cycles. This study focused on patients undergoing treatments involving split IVF-ICSI cycles, analyzing the effects of SERa on the developmental potential and clinical outcomes of sibling embryos resulting from both IVF and ICSI fertilization methods. These findings hold significant clinical implications and provide a foundation for strategic adjustments when managing SERa+ oocytes.

Our comparison of general clinical data between the two groups revealed no significant differences in age, duration of infertility, type of infertility, body mass index, and baseline levels of FSH, E_2_, LH, and P. This indicates that these baseline variables had a negligible impact on our study findings. In contrast to the SERa- cycles, the SERa+ cycles exhibited a tendency towards elevated E_2_ levels and an increased total number of oocytes retrieved on hCG day, which is consistent with previous studies (20). The occurrence of ovarian hyperstimulation may be associated with SERa since research has shown that SERa is not present in oocytes from unstimulated patients (21).The occurrence of SERa is positively correlated with E_2_ levels on the day of hCG administration, and it is widely accepted that the emergence of SERa is associated with elevated E_2_ levels (22). However, current research investigating whether increased E_2_ levels directly lead to the occurrence of SERa remains limited. A recent study examining the potential impact of aromatase inhibitor protocols on reducing SERa incidence in oocytes (23) found that these inhibitors did not significantly decrease the occurrence of SERa.

This suggests that elevated E_2_ levels may not be the primary cause of SERa; rather, its occurrence could result from a combination of inherent patient factors and ovarian stimulation. Consequently, further investigation into possible predictive factors for SERa occurrence is warranted. The primary function of smooth endoplasmic reticulum involves calcium storage and release, which are essential during processes such as oocyte activation, fertilization, and energy accumulation (24).The cytoplasmic anomaly SERa may disrupt calcium storage and oscillation during fertilization. Previous studies have reported significantly reduced fertilization rates in SERa+ cycles (25). However, other investigations, including our own, found no significant differences in total fertilization rates or normal fertilization rates between SERa+ and SERa- cycles (26). Notably, the SERa+ cycles exhibited a trend toward increased rates of high-quality Day 3 embryos, particularly ICSI, while no such differences were observed in conventional IVF. A cohort study (27) has indicated significantly lower rates of high-quality embryos in SERa+ cycles compared to their SERa- counterparts, although another study reported no differences (26). The inconsistencies among these studies regarding high-quality embryo rates may stem from non-uniform definitions of what constitutes a high-quality embryo or from small sample sizes, thus necessitating further research. Concerning the impact of SERa on blastocyst development, existing literature suggests that SERa significantly influences both blastocyst quality and developmental speed, leading to a reduction in the blastocyst formation rate (28). Our study did not reveal any significant differences between the two groups concerning overall blastocyst formation rates, high-quality blastocyst rates, or available blastocyst rates—regardless of whether IVF or ICSI was employed—consistent with recent findings (26). Some studies propose that the presence of SERa does not hinder ongoing blastocyst development nor interfere with the formation rates of high-quality embryos or affect euploidy and aneuploidy ratios (20, 30).

Our findings are consistent with several studies, indicating no significant differences between the two groups in terms of βhCG positivity rates, clinical pregnancy rates, implantation rates, early miscarriage rates, live birth rates, preterm birth rates, as well as the heights and weights of newborns (29). Embryos derived from SERa+ oocytes have the potential to develop into normal and healthy newborns. Furthermore, there is no definitive negative correlation observed between SERa+ oocytes and cycles concerning embryology, clinical outcomes, or newborn results. The question of whether SERa adversely affects embryonic developmental potential and clinical outcomes remains a topic of debate. Additionally, while our study did not identify any birth defects among live births in either group, recent meta-analyses (31) suggest that SERa+ cycles/oocytes may carry a potential risk for an increased incidence of major birth defects.

This study presents several limitations. As a retrospective analysis, it is inherently subject to biases and cannot adequately control for participant heterogeneity. The number of embryos derived from SERa+ oocytes in this study was relatively small, resulting in a limited sample size. Our investigation concentrated on SERa+ cycles rather than SERa+ oocytes; therefore, caution should be exercised when interpreting the results of this study. Future research with larger sample sizes and prospective designs is essential for validating our findings. Additionally, further long-term follow-up regarding clinical outcomes and newborns resulting from embryos derived from SERa+ oocyte transfers is necessary to evaluate the potential for developmental abnormalities.

Given the clinical significance of SERa+ oocytes, we propose establishing an international multicenter registry to systematically track outcomes of embryos derived from these oocytes. Such a registry would enable: (1) standardized data collection on fertilization rates, embryo quality, and pregnancy outcomes; (2) correlation of SERa+ morphology with genetic and epigenetic profiles; and (3) development of evidence-based guidelines for clinical management.

In conclusion, this study indicates that SERa is associated with hormone levels in patients undergoing assisted reproductive technology; however, it does not appear to influence embryonic development or clinical outcomes. Consequently, discarding SERa oocytes may not represent the most ethical approach. The avoidance of wastage of oocytes and embryos remains a persistent concern in daily IVF practice. Nevertheless, current conclusions regarding the developmental and clinical outcomes of embryos derived from SERa are inconsistent. Caution is warranted when transferring embryos originating from SERa oocytes in assisted reproductive treatments, highlighting the need for large-scale, multicenter data studies. Further investigations into the causes and mechanisms underlying SERa formation in oocytes are essential to provide evidence that supports decision-making during clinical embryo transfers, ultimately enhancing clinical outcomes for patients experiencing infertility.

## Data Availability

The raw data supporting the conclusions of this article will be made available by the authors, without undue reservation.

## References

[1] Brown AM McCarthy HE. The Effect of CoQ10 supplementation on ART treatment and oocyte quality in older women. Hum Fertil (Camb) (2023) 26:1544–52. doi: 10.1080/14647273.2023.2194554 37102567

[2] Anagnostopoulou C Rosas IM Singh N Gugnani N Chockalingham A Singh K. Oocyte quality and embryo selection strategies: a review for the embryologists, by the embryologists. Rev Panminerva Med. (2022) 64:171–84. doi: 10.23736/S0031-0808.22.04680-8 35179016

[3] Serhal PF Ranieri DM Kinis A Marchant S Davies M Khadum IM. Oocyte morphology predicts outcome of intracytoplasmic sperm injection. Hum Reprod. (1997) 12:1267–70. doi: 10.1093/humrep/12.6.1267 9222015

[4] Ferreux L Sallem A Chargui A Gille AS Bourdon M Maignien C. Is it time to reconsider how to manage oocytes affected by smooth endoplasmic reticulum aggregates? Rev Hum Reprod. (2019) 34:591–600. doi: 10.1093/humrep/dez010 30805638

[5] Machaca K. Increased sensitivity and clustering of elementary Ca2+ release events during oocyte maturation. Dev Biol. (2004) 275:170–82. doi: 10.1016/j.ydbio.2004.08.004 15464580

[6] Ozil J-P Markoulaki S Toth S Matson S Banrezes B Knott JG. Egg activation events are regulated by the duration of a sustained [Ca2+] cyt signal in the mouse. Dev Biol. (2005) 282:39–54. doi: 10.1016/j.ydbio.2005.02.035 15936328

[7] Otsuki J Okada A Morimoto K Nagai Y Kubo H. The relationship between pregnancy outcome and smooth endoplasmic reticulum clusters in MII human oocytes. Hum Reprod. (2004) 19:1591–7. doi: 10.1093/humrep/deh258 15180981

[8] Ebner T Moser M Shebl O Sommerguber M Tews G. Prognosis of oocytes showing aggregation of smooth endoplasmic reticulum. Reprod BioMed Online. (2008) 16:113–8. doi: 10.1016/S1472-6483(10)60563-9 18252056

[9] Akarsu C Çağlar G Vicdan K Sözen E Biberoğlu K. Smooth endoplasmic reticulum aggregations in all retrieved oocytes causing recurrent multiple anomalies: case report. Fertil Steril. (2009) 92:1496. doi: 10.1016/j.fertnstert.2009.06.048 19646689

[10] Bielanska M Leveille M. Live births from oocytes with smooth endoplasmic reticulum (SER) dysmorphism. Hum Reprod. (2011) 26:i163. doi: 10.1093/humrep/26.s1.79

[11] Alpha Scientists in Reproductive Medicine and ESHRE Special Interest Group of Embryology. The istanbul consensus workshop on embryo assessment: proceedings of an expert meeting. Hum Reprod. (2011) 26:1270–83. doi: 10.1016/j.rbmo.2011.02.001 21502182

[12] Mateizel I Van Landuyt L Tournaye H Verheyen G. Deliveries of normal healthy babies from embryos originating from oocytes showing the presence of smo oth endoplasmic reticulum aggregates. Hum Reprod. (2013) 28:2111–7. doi: 10.1093/humrep/det241 23696540

[13] Hattori H Nakamura Y Nakajo Y Araki Y Kyono K. Deliveries of babies with normal health derived from oocytes with smooth endoplasmic reticulum clusters. J Assist Reprod Genet. (2014) 31:1461–7. doi: 10.1007/s10815-014-0323-z PMC4389939 25205205

[14] Itoi F Asano Y Shimizu M Honnma Hi Murata Y. Embryological outcomes in cycles with human oocytes containing large tubular smooth endoplasmic reticulum clusters after conventional *in vitro* fertilization. Gynecol Endocrinol. (2016) 32:315–8. doi: 10.3109/09513590.2015.1115831 26607857

[15] Van Beirs N Shaw-Jackson C Rozenberg S Autin C. Policy of IVF centres towards oocytes affected by smooth endoplasmic reticulum aggregates: A multicentre survey study. J Assist Reprod Genet. (2015) 32:945–50. doi: 10.1007/s10815-015-0473-7 PMC4491080 25894687

[16] Alpha Scientists in Reproductive Medicine and ESHRE Special Interest Group of Embryology. Electronic address. The vienna consensus: report of an expert meeting on the development of ART laboratory performance indicators. Reprod BioMed Online. (2017) 35:494–510. doi: 10.1016/j.rbmo.2017.06.015 28784335

[17] Peng N Ma S Li C. Intracytoplasmic sperm injection may not improve clinical outcomes despite its positive effect on embryo results: A retrospective analysis of 1130 half-ICSI treatments. Front Endocrinol (Lausanne). (2022) 13:877471. doi: 10.3389/fendo.2022.877471 35784567 PMC9240197

[18] Jiang L Qian Y Chen X Ji X Ou S Li R. Effect of early rescue ICSI and split IVF-ICSI in preventing low fertilization rate during the first ART cycle: A real-world retrospective cohort study. Reprod Med Biol. (2021) 21:e12420. doi: 10.1002/rmb2.12420 34934401 PMC8656193

[19] Goswami G Gouri MD. Relevance of split *in vitro* fertilization-intracytoplasmic sperm injection method of insemination in normozoospermic and mildly oligospermic men: A retrospective study. J Hum Reprod Sci. (2020) 13:145–9. doi: 10.4103/jhrs.JHRS_19_19 PMC7394098 32792764

[20] Xu J Yang Li Chen Z-H. Oocytes with smooth endoplasmic reticulum aggregates are not associated with impaired reproductive outcomes: A matched retrospective cohort study. Front Endocrinol (Lausanne). (2021) 12:688967. doi: 10.3389/fendo.2021.688967 34512544 PMC8426629

[21] Nikiforov D Cadenas Jesús Mamsen LS Wakimoto Y Kristensen SG Pors SE. Clusters of smooth endoplasmic reticulum are absent in oocytes from unstimulated women. Reprod BioMed Online. (2021) 43:26–32. doi: 10.1016/j.rbmo.2021.03.007 34006484

[22] Siddhartha N Reddy NS Pandurangi M Tamizharasi M Radha V Kanimozhi K. Correlation of serum estradiol level on the day of ovulation trigger with the reproductive outcome of intracytoplasmic sperm injection. J Hum Reprod Sci. (2016) 9:23–7. doi: 10.4103/0974-1208.178631 PMC4817283 27110074

[23] Saito H Otsuki J Takahashi H Hirata R Habara T Hayashi N. A higher incidence of smooth endoplasmic reticulum clusters with aromatase inhibitors. Reprod Med Biol. (2019) 18:384–9. doi: 10.1002/rmb2.12296 PMC6780026 31607799

[24] Van Blerkom J. Mitochondrial function in the human oocyte and embryo and their role in developmental competence. Mitochondrion. (2011) 11:797–813. doi: 10.1016/j.mito.2010.09.012 20933103

[25] Sa R Cunha M Silva J Luís A Oliveira C Silva JT. Ultrastructure of tubular smooth endoplasmic reticulum aggregates in human metaphase II oocytes and clinical implications. Fertil Steril. (2011) 96:143–9. doi: 10.1016/j.fertnstert.2011.04.088 21621206

[26] Kong P Pan J Liang S Yin M Teng X. Blastocysts originated from oocytes with smooth endoplasmic reticulum aggregates have a reduced euploidy rate: a retrospective cohort study. Front Endocrinol (Lausanne). (2024) :1425578. doi: 10.3389/fendo.2024.1425578 39403582 PMC11471620

[27] Fang T Yu W Ou S. The impact of oocytes containing smooth endoplasmic reticulum aggregates on assisted reproductive outcomes: a cohort study. BMC Pregnancy Childbirth. (2022) 22:838. doi: 10.1186/s12884-022-05141-9 36376855 PMC9664725

[28] Wang X Xiao Y Sun Z Zhen J Yu Q. Smooth endoplasmic reticulum clusters in oocytes from patients who received intracytoplasmic sperm injections negatively affect blastocyst quality and speed of blastocyst development. Affiliations Expand. (2021) 12:732547. doi: 10.3389/fphys.2021.732547 PMC8695965 34955873

[29] Wang M Gao L Yang Q Long R Zhang Y Jin L. Does smooth endoplasmic reticulum aggregation in oocytes impact the chromosome aneuploidy of the subsequent embryos? A propensity score matching study. J Ovarian Res. (2023) 16:59. doi: 10.1186/s13048-023-01135-z 36959673 PMC10037775

[30] Chiu CS-C Hung T-Y Lin M-H Lee RK-K Weng YW Hwu YM. Metaphase II (MII) human oocytes with smooth endoplasmic reticulum clusters do not affect blastocyst euploid rate. Taiwan J Obstet Gynecol. (2022) 61:585–9. doi: 10.1016/j.tjog.2021.03.044 35779904

[31] Long R Wang M Yang Q Zhang Y Gao L Jin L. Smooth endoplasmic reticulum aggregates in oocytes associated with increased risk of neonatal birth defects: A meta-analysis. Acta Obstet Gynecol Scand. (2024) 103:2163–70. doi: 10.1111/aogs.14910 PMC11502435 38961609

